# Development of the Malocclusion Impact Questionnaire (MIQ) to measure the oral health-related quality of life of young people with malocclusion: part 2 – cross-sectional validation

**DOI:** 10.1080/14653125.2015.1114223

**Published:** 2016-01-08

**Authors:** Philip E. Benson, Susan J. Cunningham, Nahush Shah, Fiona Gilchrist, Sarah R. Baker, Samantha J. Hodges, Zoe Marshman

**Affiliations:** ^a^ Academic Unit of Oral Health & Development, School of Clinical Dentistry, University of Sheffield, UK; ^b^ Orthodontic Department, University College London Eastman Dental Institute, UK; ^c^ Academic Unit of Dental Public Health, School of Clinical Dentistry, University of Sheffield, UK

**Keywords:** Impact, malocclusion, oral health quality of life, orthodontics, questionnaire

## Abstract

**Objective**: To test the items, identified through qualitative inquiry that might form the basis of a new Malocclusion Impact Questionnaire (MIQ) to measure the oral health-related quality of life (OHQoL) of young people with malocclusion. **Methods**: Piloting with 13 young people reduced the number of items from 37 to 28. Cross-sectional testing involved a convenience sample aged 10–16 years, attending the Orthodontic Department of the Charles Clifford Dental Hospital, Sheffield. The fit and function of the initial MIQ questions were examined using item response theory. **Results**: 184 participants (113 females; 71 males) completed a questionnaire (response 85%), seven participants were excluded due to missing responses. The mean age of participants was 12·9 years (SD 1·4) and they had a wide range of malocclusions. The majority were White British (67·4%). Data from 47 participants were used to analyse test–retest reliability. Rasch analysis was undertaken, which further reduced the number of items in the questionnaire from 28 to 17. Unidimensionality of the scale was confirmed. The analysis also identified that the original 5-point response scale could be reduced to three points. The new measure demonstrated good criterion validity (*r* = 0·751; *P* < 0·001) and construct validity with the two global questions (‘Overall bother’ *ρ* = 0·733 and ‘Life overall’ *ρ* = 0·701). Internal consistency (Cronbach's alpha = 0·906) and test–retest reliability Intraclass correlation coefficient (ICC = 0·78; 95% CI 0·61–0·88) were also good. **Conclusion**: Cross-sectional testing has shown the new MIQ to be both valid and reliable. Further evaluation is required to confirm the generalisability as well as the ability of the new measure to detect change over time (responsiveness).

## Introduction

Doubts have been expressed about the suitability of some of the current generic measures for assessing OHQoL in young people seeking orthodontic treatment (Marshman *et al*., 2010). In Part 1 of this report, we described the first two stages of developing a Malocclusion Impact Questionnaire (MIQ) to measure the OHQoL of young people with malocclusion, which involved:

- Specifying measurement goals: using descriptors appropriate for measuring the oral health-related quality of life (HRQoL) in adolescents with malocclusion;
- Item generation: populating the measure with suitable items on the basis of qualitative inquiry.

In this report, we describe the further development of MIQ involving:

- Questionnaire formatting: including selecting the appropriate response options, wording and language to avoid leading and biased questions;
- Item reduction: reducing items on the basis of their intensity, frequency and importance;
- Cross-sectional testing to determine validity, internal consistency/reliability and test–retest repeatability.

## Methods

Ethics approval from the Proportionate Review Sub-committee of the NRES Committee North East – Sunderland Research Ethics Committee (24 November 2011; REC Reference 11/NE/0359) covered the cross-sectional validation at Sheffield.

### Questionnaire formatting

The initial MIQ was constructed based on the themes derived from the framework analysis and consisted of 37 questions broadly divided into three sections:

- How I feel about the way my teeth look;
- How my teeth affect my life;
- Eating and the health of my teeth, including knocks and bangs to my teeth.

The response format for MIQ was chosen following previous work suggesting that the severity or intensity of the malocclusion impact was more important to young people than the frequency (Marshman *et al*., 2010). The wording for the response options was based upon the work carried out by Stevens, when she interviewed young people with a wide range of acute and chronic health conditions whilst developing a new preference-based measure of HRQoL (Stevens, 2009). Stevens found that common adverbs and adverbial phrases used by the young people to describe their HRQoL were ‘a little bit’, ‘a bit’, ‘quite’ and ‘very’; therefore, these words were incorporated into a 5-point scale to describe the severity of their impact.

An initial pilot of the MIQ was undertaken with eight young people at the Eastman and five young people in Sheffield, who were observed whilst completing the questionnaire and interviewed about the wording, clarity, readability, acceptability and interpretation of each question. Participants were also invited to comment on the questionnaire as a whole. Changes to the wording of items, instructions and response formats were made following each interview and the revised instrument shown to the next participant. The 37 items in the initial MIQ were reduced to 28 following this pilot testing. A Flesch Kincaid reading score™ of the questionnaire showed that it was acceptable for an 11 year old to read.

### Cross-sectional evaluation

A further convenience sample of participants attending for a new patient appointment was recruited from the Orthodontic Department of the Charles Clifford Dental Hospital, Sheffield. The intended sample size was between 150 and 200, which is usually considered sufficient for an appropriate statistical analysis (Guyatt *et al*., 1986).

The inclusion criteria were young people:

- aged 10–16 years;
- either gender and any ethnic group;
- who described themselves as ‘needing a brace’.

The exclusion criteria were young people with a:

- history of previous orthodontic treatment;
- severe skeletal discrepancy or a cleft of the lip and/or palate;
- complex medical history or learning disability that would impair understanding of the measure.

Potential participants and their parents were approached in the Orthodontic Department at their first appointment as a new patient. The young people were asked ‘Do you think you need a brace?’ If they replied ‘Yes’, then they were invited to take part in the study, the purpose of which was described in general terms. The young people and their parents were given separate written information sheets, as well as the questionnaire. The young people were encouraged to complete the questionnaire on their own and return it at their initial visit, for example, whilst waiting to have diagnostic radiographs. If this was not possible then they were asked to take the questionnaire away, complete it at their convenience and return it in a pre-paid envelope, which was provided.

Each questionnaire consisted of a front sheet, which was detached and completed by the clinician, containing the participant's allocated study number and summary details of their occlusion. The participant was given the rest of the measure, with their participant study number, to self-complete, starting with their demographic details (age, gender, ethnicity), followed by the short form of the Child Perceptions Questionnaire (CPQ_11–14_-ISF16), a generic measure of OHQoL (Jokovic *et al*., 2006), then the 28 item MIQ. The last section of the measure contained three global questions about how they would rate the health of their mouth, teeth and gums; how much their teeth affected their life overall and how much their teeth bothered them (Jokovic *et al*., 2002).

The last question asked each participant if they would be prepared to complete the questionnaire again. Those participants who ticked the box were sent a new questionnaire, to their home address, after at least 2 weeks, with a pre-paid return envelope. The start of the repeat questionnaire was modified to ask if anything had changed since they had last completed it, i.e. they had had some teeth extracted or a brace fitted. Only data from those who indicated no change were analysed.

### Item reduction

Data were entered onto an Excel spreadsheet (v 2010, Microsoft Corp, Washington, USA). Where a participant missed more than eight items, the entire questionnaire was excluded from the analysis. When fewer than eight responses were missing, each absent value was substituted with the mean for the individual (Shrive *et al*., 2006).

The fit and function of the initial MIQ questions were examined using an item response theory (IRT) Rasch model. Rasch analysis was originally used in educational testing, but more recently has been used in the development and validation of patient-reported outcome measures (Batcho *et al*., 2012; Chien *et al*., 2014; Shelton *et al*., 2015). Formal testing of a scale against a mathematical model assesses how well the participant responses fit the model (Rasch, 1960). These expectations are based on the probabilistic form of Guttman scaling (Guttman, 1950; Smith, 2000). According to this method, the items chosen for the final measure should be unidimensional, free from differential item functioning (DIF), i.e. they function in the same way across groups, and fit the model expectations (Tennant *et al*., 2007). The overall score can then be expressed in logits (log odds probability units), thus converting the ordinal raw scores to an interval scale from which accurate change scores can be calculated.

The measure was tested with the unrestricted or partial credit model, using the method suggested by Tennant *et al*. (2007) involving:

1. Category discrimination: This analyses response patterns to assess whether participants are able to discriminate between the different response options. Where these are disordered, adjacent categories can be collapsed to reduce the number of response options.
2. Local dependency was deemed to be present if residual correlations were greater than 0·2 above the average residual correlation (Kersten *et al*., 2014).
3. DIF was analysed by age (10–13 years and 14–16 years) and gender.
4. Item fit to the model: If the data fit the Rasch model, each item and person fit residual should be within the range ±2·5 and the mean item and person fit statistics should be close to zero with a standard deviation of one (Kersten *et al*., 2014). Finally, the individual items and summary chi-square interaction statistics should be non-significant (>0·05), although these are subject to Bonferroni adjustment based on the number of items. Strict unidimensionality was then examined using an independent t-test on two subsets of items identified using principal component analysis of the item residuals.
5. Reliability: reliability was evaluated using the Person Separation Index (PSI). This is equivalent to Cronbach's alpha, however the logit value is used instead of the raw score. It is interpreted in the same manner, i.e. a value of greater than 0·7 is recommended.

Once a unidimensional scale had been achieved, a transformation from raw score to interval data was undertaken. All further analyses were based on the scale created from this analysis. The Rasch analysis was undertaken using RUMM2030 (RUMM Laboratory Pty Ltd, WA, Australia).

### Cross-sectional testing

The response format for MIQ consisted of a 5-point severity scale based on the Child Health Utility 9D index (CHU9D), which is a generic child HRQoL (Stevens, 2009), i.e. ‘don't’ or ‘am not’, ‘a little bit’, ‘a bit’, ‘quite a lot’ and ‘very much’. Each item was scored 0–4, the order depending on whether the stem was positively worded (‘Happy’, ‘Good looking’, ‘Confident’) or negatively worded (‘Nervous’, ‘Shy’). Again the scores for each item are added together to obtain a total score, higher scores indicating poorer OHQoL.

Criterion validity was assessed by examining the correlation between the total scores of MIQ with the total scores of the accepted gold standard (CPQ_11–14_-ISF16) using a Pearson product correlation coefficient. CPQ_11–14_-ISF16 is organised into four subscales (oral symptoms, functional limitations, emotional well-being and social well-being), with a frequency response format, which is scored 0 = ‘Never’, 1 = ‘Once/twice’, 2 = ‘Sometimes’, 3 = Often and 4 = ‘Everyday/almost everyday’. The scores for each item are added together to obtain a total score. The minimum possible score is 0 and maximum possible score is 64, with higher scores indicating poorer OHQoL.

Construct validity was assessed by examining the correlation between the total scores with those of the global questions using a Spearman's rank correlation. The global rating of oral health was scored from 0 = ‘Excellent’ to 4 = ‘Poor’. The global rating of impact on life overall was scored from 0 = ‘Not at all’ to 4 = ‘Very much’. The rating of satisfaction with the appearance of their own teeth was scored from 0 = ‘Very satisfied’ to 4 = ‘Very dissatisfied’.

Cronbach's alpha was used to test the internal consistency/reliability and intraclass correlation coefficients calculated by the one-way analysis of variance random effects parallel model for test–retest reliability. Statistical tests were undertaken using SPSS (v20 IBM Corp., NY, USA).

## Results

### Descriptive data

The recruitment period for the validation study was November 2013 to September 2014. During this time, 216 young people were invited to take part and 184 completed questionnaires were received (response 85%).

The demographic and clinical information for the included participants is shown in Table 1. There were 113 females (61%) and 71 males (39%), with a mean age of 12·9 years (SD 1·4). There were ethnicity data for 183 participants and 123 described themselves as White British (67·2%). Table 1 shows that the participants had a wide range of malocclusions, with overjets ranging from −4 to 13 mm and 49·5% had moderate-to-severe crowding in the upper arch, mainly in the upper labial segment (85·3%). Over one quarter of participants (*n* = 53; 28·8%) had at least one developmentally absent tooth.

**Table 1:** Demographics and clinical data for the included participants (*N* = 184).

		*N*	%
Gender	Male	71	39
	Female	113	61
Age (years)	10	11	6·0
	11	21	11·4
	12	40	21·7
	13	44	23·9
	14	43	23·4
	15	23	12·5
	16	2	1·1
Ethnicity^a^	White	123	67·2
	Black British	39	21·3
	Black African	7	3·8
	Mixed	4	2·2
	Black other	1	0·5
	Pakistani	7	3·8
	Other	2	1·1
Incisor relationship^b^	Class I	55	30·1
	Class II division 1	66	36·1
	Class II division 2	24	13·1
	Class II intermediate	7	3·8
	Class III	31	16·9
Upper arch	Spaced	43	23·4
	No crowding or mild (0–4 mm)	50	27·2
	Moderate (5–8 mm)	52	28·3
	Severe (>8 mm)	39	21·2
Lower arch^c^	Spaced	19	10·4
	No crowding or mild (0–4 mm)	114	62·6
	Moderate (5–8 mm)	34	18·7
	Severe (>8 mm)	15	8·2

^a^Data missing for 1 participant.

^b^One participant had missing lower incisors and no judgement was made of the incisor relationship or OJ measurement.

^c^Data missing for two participants.

Out of 184 participants, there were complete CPQ_11–14_-ISF16 data for 172 and complete MIQ data for 166 participants. Eight participants had one missing CPQ response, three had two missing CPQ responses and one had three missing CPQ responses. Eight participants had one missing MIQ response, one had two missing MIQ responses, one had three missing MIQ responses and one had four missing MIQ responses. The missing data were replaced by the mean values for these participants. Seven participants had more than eight missing MIQ responses due to a printing error and the data from these participants were excluded; therefore, CPQ and MIQ data from 177 participants were analysed.

### Item reduction

The initial scale showed significant misfit to the model (Table 2). All but one item had disordered thresholds, indicating that the response categories were not functioning as expected; therefore, the 5-point scale was changed to a 3-point scale by collapsing the 2nd, 3rd and 4th categories. One item demonstrated DIF by age group (‘Being teased’) and was therefore removed. Ten items (‘Embarrassed’, ‘Having my photograph taken’, ‘People laughing at me’, ‘People saying nasty things about my teeth’, ‘Doing well at school’, ‘Getting a job’, ‘Keeping my teeth clean’, ‘Keeping my teeth healthy, ‘Food getting stuck and causing problems with my teeth’ and ‘Damaging my teeth during activities or sports’) displayed misfit to the model or high residual correlations and were also removed from further analysis. Removal of these eleven items resulted in good fit to the model. There remained some residual correlations greater than 0·2. These were paired items where some correlation might be expected (‘Happy’ and ‘Good looking’; ‘Sad’ and ‘Bullied’; ‘Smile’ and ‘Laugh’; ‘Making friends’ and ‘Fitting in with friends’). Removal of these items did not improve the fit, therefore they were retained. Five participants demonstrated misfit to the model. Their raw data were examined and no obvious reason for the misfits was found; however, removal of their data resulted in improved fit statistics.

**Table 2:** Fit to the Rasch model.

Analysis name	Item residual		Person residual		Chi-square		Reliability	Unidimensionality	
	Mean	SD	Mean	SD	Value (df)	*P*	PSI	Proportion of tests >5%	Lower 95% CI proportion
Initial analysis	−0·10	1·53	−0·16	1·31	238 (56)	<0·001	0·92	31·4%%	0·28
Rescored to 3-point scale	−0·22	1·47	−0·28	1·47	140 (56)	<0·001	0·91	25·0%	0·22
Remove misfitting/highly correlated items/DIF	−0·32	0·76	−0·33	1·11	48 (34)	0·06	0·88	7·56%	0·04
Remove five misfitting persons	−0·29	0·79	−0·30	1·02	48 (34)	0·06	0·89	6·59%	0·03
Ideal	0	1	0	1		>0·0006^a^	>0·7	<5%	≤0·05

^a^Bonferroni adjusted for 17 items.

df = degrees of freedom; CI = confidence interval.

Overall fit statistics at each stage of analysis are shown in Table 2, along with the ideal statistics. Table 3 shows the item fit statistics for the 17 retained items, which are ordered from ‘easiest’ (‘Feeling happy’) to ‘most difficult’ (‘Fitting in with friends’). The mean person location is −1·30 when the items are centred on zero. This demonstrates that the scale is targeted to a population with more impacts than the participants in this study.

**Table 3:** Item fit statistics ordered by location.

Item	Location	Standard error	Fit residual	Degrees of freedom	Chi-square>
Happy	−2·40	0·17	−1·23	154·27	0·59
Good looking	−2·37	0·17	−0·36	152·41	4·92
Confident	−1·52	0·16	−1·42	153·34	4·96
Smile	−1·03	0·15	0·32	154·27	2·55
Seeing photographs of myself	−0·60	0·14	−0·30	154·27	0·14
Normal	−0·59	0·16	−0·63	153·34	2·66
Other people have nicer teeth than me	−0·52	0·15	−0·39	154·27	0·76
Laugh	−0·25	0·15	−0·99	154·27	5·49
Shy	0·48	0·17	−1·08	154·27	4·54
Cover my teeth with my hand when I smile	0·50	0·16	−0·34	154·27	0·12
Nervous	0·68	0·17	0·16	154·27	0·74
Talking in public	0·76	0·17	−0·19	154·27	0·17
Being bullied	0·90	0·17	0·53	153·34	1·72
Biting some foods	1·12	0·18	1·94	154·27	12·79
Sad	1·20	0·18	−0·73	154·27	2·00
Making friends	1·72	0·20	−0·05	154·27	0·53
Fitting in with friends	1·94	0·20	−0·22	154·27	2·95
Ideal			≤±2·5		>0·0006^a^

^a^Bonferroni adjusted for 17 items.

**Figure 1:**
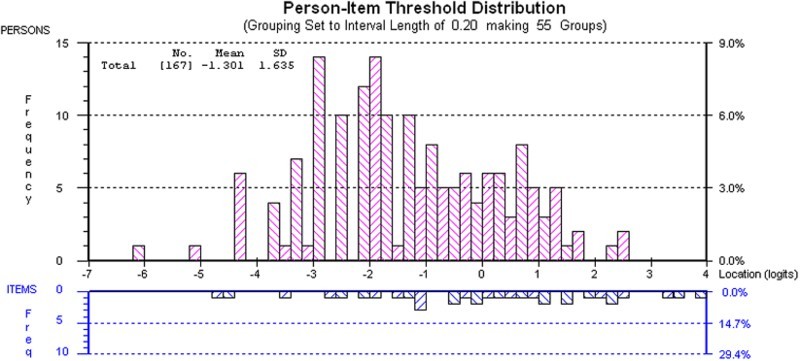
Targeting of MIQ. The upper section of the graph shows the distribution of participants and the lower part the distributions of thresholds (category transitions) of the items. The *x*-axes display the location (severity of impact) of participants and the item location (difficulty) of the item thresholds. The *y*-axes show the frequency of item thresholds and participants

Figure 1 shows the person-item threshold map which indicates that participants are distributed in a similar pattern to the items and that the items measure the impacts of malocclusion along the construct from least to most. As the items fit the Rasch model, a transformation from the raw score to interval scaling is shown in Table 4.

**Table 4:** Raw (ordinal) score to interval score transformation.

Raw score	Interval score	Raw score	Interval score
0	0·00	17	18·08
1	2·96	18	18·65
2	5·23	19	19·23
3	6·96	20	19·80
4	8·37	21	20·39
5	9·55	22	20·98
6	10·57	23	21·59
7	11·48	24	22·22
8	12·30	25	22·87
9	13·06	26	23·56
10	13·78	27	24·29
11	14·45	28	25·08
12	15·10	29	25·94
13	15·72	30	26·92
14	16·33	31	28·06
15	16·92	32	29·46
16	17·50	33	31·38
		34	34·00

### Validity testing

Table 5 shows the descriptive data for the domain and total scores for CPQ_11–14_-ISF16. There were no floor (minimum score 0) or ceiling effects (maximum score 64). Table 5 also shows the descriptive data for the total MIQ scores collapsed into three response options, as suggested by the Rasch analysis. There were no ceiling effects (maximum score 34); however, one participant did score 0, suggesting a floor effect; but when they repeated the questionnaire they had a very high total score indicating that they might have misread the instructions the first time around. Excluding this participant there were five participants with a total MIQ score of less than 5, compared with 16 participants with a total CPQ_11–14_-ISF16 score of less than 5. This suggests that floor effects might be more of an issue with the generic, rather than the condition-specific measure, which is to be expected.

**Table 5:** Descriptive data for the questionnaire responses.

	Domain	Median	Mean	SD	Min	Max
CPQ_11–14_-ISF16 (*N* = 184)	Oral symptoms	4	4·3	2·3	0	10
	Functional limitations	2	3·2	2·8	0	11
	Emotional well-being	4	5·0	4·3	0	16
	Social well-being	3	3·3	3·2	0	15
	Total score	14	15·8	9·5	1	47
MIQ_10–16_ (*N* = 177)	Total score	10	11·6	6·5	0	28

**Figure 2:**
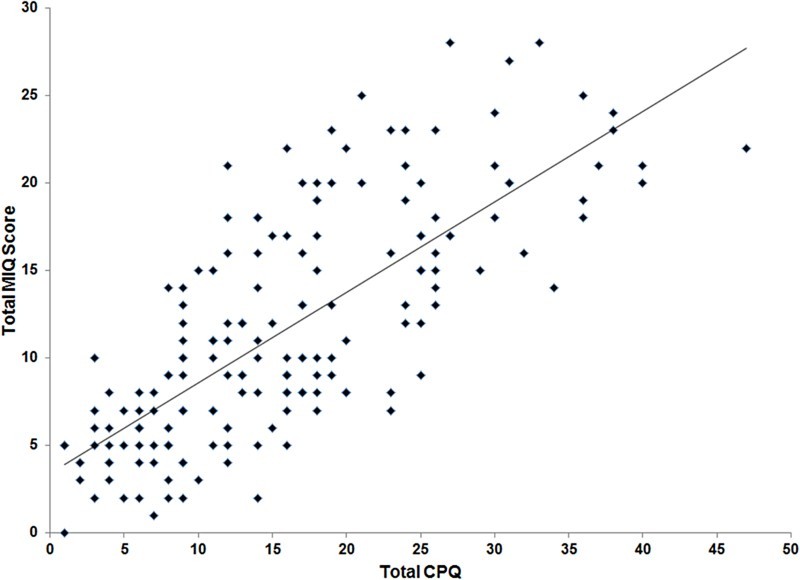
Scatterplot of the total CPQ_11–14_-ISF16 and total MIQ scores

**Figure 3:**
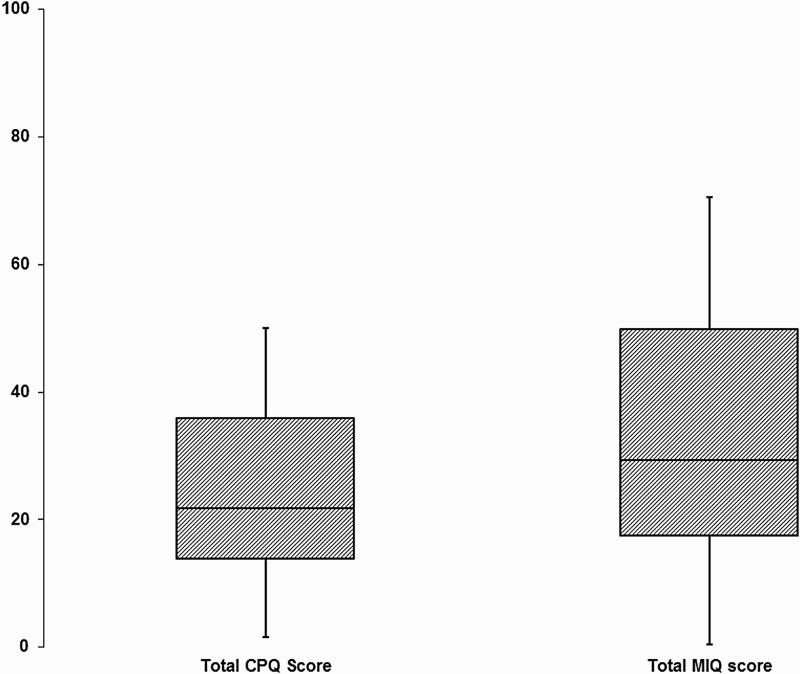
Boxplots of the standardised total scores for CPQ_11–14_-ISF16 and MIQ

A scatterplot between the total scores for MIQ and the accepted gold standard measure CPQ_11–14_-ISF16 is shown in Figure 2. The correlation between the two total scores was high (*r* = 0·751; *P* < 0·001) suggesting that MIQ showed excellent criterion validity with CPQ_11–14_-ISF16. The two measures have similar scoring methods; however, to investigate their ability to discriminate between individuals or timepoints the scores were standardised to a scale of 0–100 (CPQ scores × 100/max score of 64; MIQ scores × 100/max score of 34). These standardised scores were plotted as boxplots (Figure 3) and indicate that there was a greater spread for the responses to MIQ, suggesting enhanced discrimination.

The correlations between the three global questions and the two measures are shown in Table 6. The correlations between both measures of OHQoL and the global question ‘Overall, how much do your teeth bother you?’ were high indicating good validity for this construct. MIQ also had a high correlation with the question ‘Overall, how much do your teeth affect your life?’. The correlations between both measures of OHQoL and the global question ‘Overall, how would you rate your teeth?’ were much lower, indicating a poor relationship with this construct.

**Table 6:** Spearman correlation coefficients (*ρ*) between CPQ11–14-ISF16 and the three global questions and between the condition-specific measure and the three global questions.

	Oral health	How bothered	Life overall
CPQ_11–14_-ISF16	0·270	0·722	0·589
MIQ_10–16_	0·236	0·733	0·701

### Reliability testing

The Cronbach's alpha score for CPQ_11–14_-ISF16 was 0·841 and for MIQ was 0·906 confirming that the internal consistency reliability for both questionnaires is high.

The number of participants who indicated that they would be prepared to repeat the questionnaire was 134. These were all sent repeat questionnaires and 56 responded (response rate 42%); however, eight participants indicated that they had had their brace fitted or some extractions carried out since they first completed the questionnaire, so were excluded from the analysis. The MIQ data from one participant was also excluded, as they had more than eight items missing from their first questionnaire due to a printing error; therefore, data from 48 participants were used to analyse test–retest repeatability for CPQ_11–14_-ISF16 and the data from 47 participants were used to analyse test–retest repeatability for MIQ.

The intraclass correlation coefficients for the repeat total CPQ_11–14_-ISF16 scores was 0·86 (95% CI 0·75–0·92) and for the repeat MIQ scores was 0·78 (95% CI 0·61–0·88), indicating good repeatability.

## Discussion

The aim of this study was to test the validity, reliability and repeatability of a previously developed condition-specific measure of OHQoL in young people with malocclusion (MIQ). A new measure is required because there are concerns that the current generic measures of OHQoL fail to capture all of the issues experienced by young people with malocclusion (Marshman *et al*., 2010).

The questionnaire was developed using the stages described by Guyatt *et al*. (1986) and Juniper *et al*. (1996) to ensure that it is appropriate and relevant to young people with malocclusion. For content validity, the items were chosen following open-ended, one-to-one interviews with young people discussing the effect that malocclusion had on their day-to-day life and describing why they considered it necessary to seek orthodontic treatment. For appropriate wording, layout and face validity the questionnaire was repeatedly shown and discussed with young people.

The response format for the questionnaire was chosen following previous work suggesting that the severity or intensity of the malocclusion impact was more important to young people than the frequency (Marshman *et al*., 2010). Some of those interviewed expressed the view that they might have only one or even no experience of a situation, for example bullying about their teeth, but that the anticipated concern was very great. On the other hand, they might experience frequent episodes of an event, such as teasing, which was not considered to be serious and did not concern them. In the original report outlining the development of the CPQ_11–14_, the authors do not explain why they decided to use a frequency response format for the final questionnaire (Jokovic *et al*., 2002).

CPQ was developed using classical test theory and an item-impact approach. In this method, items elicited from qualitative interviews are given to groups of patients who report whether they experience the problem and how much it bothers them. An item-impact score is calculated by multiplying the prevalence of the problem by its mean ‘bother rating’. The items are ranked and those above the median are generally included in the questionnaire. The danger of an item-impact approach is the possible elimination of high impact, but low prevalence items, that are important to a minority of people. This results in the formation of a group-centred questionnaire, which may not be suitable for monitoring individual patients (Guyatt *et al*., 1986). The Rasch model of analysis alleviates this limitation. It is based on IRT and was originally used in educational testing. It is increasingly used in the development and validation of patient-reported outcome measures (Batcho *et al*., 2012; Chien *et al*., 2014; Shelton *et al*., 2015).

The wording for the response format was based upon the work carried out by Stevens (2009). The young people in Steven's study were younger (aged 7–11 years) than the participants in the current study; however, the wording was found to work well when tested with young people aged 10–15 years.

The responses of the young people to the Child Perceptions Questionnaire were compared with the MIQ to test criterion validity. The CPQ was used for this purpose, in spite of some reservations about the validity of the former in young people with malocclusion, because it is a commonly used generic measure of OHQoL. There was a high correlation between the responses from the two questionnaires, which suggests that the new measure demonstrates good criterion validity with the previously validated measure.

Three global questions were used to evaluate the construct validity of the measure. Both measures had good validity with the construct expressing how much the young people were bothered by their teeth, a term frequently used by participants in a previous study (Marshman *et al*., 2010). The MIQ also had a high correlation with the global question ‘Overall, how much do your teeth affect your life?’ The correlation between this rating and the total CPQ_11–14_-ISF16 scores was smaller; however, this was a higher value than in the original validation study of CPQ (*ρ* = 0·40), albeit to a slightly different global question ‘How much does the condition of your teeth, lips, jaws or mouth affect your life overall?’ (Jokovic *et al*., 2002). The correlations between the global question ‘Overall, how would you rate your teeth?’ and both measures were low and similar to the value obtained by Jokovic *et al.*, (*ρ* = 0·23), but again the question was slightly different (‘Would you say that the health of your teeth, lips, jaws and mouth is … ?’) (Jokovic *et al*., 2002). A large majority of participants (68%) described the health of their teeth as ‘Excellent’, ‘Very good’ or ‘Good’ and only 7·4% described the health of their teeth as ‘Poor’. In contrast a similar proportion of participants (68·6%) were ‘Somewhat’, ‘Quite a bit’ or ‘Very much’ bothered about their teeth. This suggests that most participants did not equate their malocclusion with poor health of their teeth. Indeed it is expected that patients referred for an orthodontic opinion would be regular attenders to the dentist and have any dental disease under control before they are referred. Perhaps this global question is not appropriate in the context of potential orthodontic patients.

### Strengths of the study

The initial development of MIQ was undertaken using qualitative interviews to involve young people with malocclusion. Young people were consulted at each stage to further amend and refine the measure resulting in good face and content validity. Data collected during the cross-sectional validation showed that the young people had a range of malocclusions that are representative of the adolescent population who seek orthodontic treatment.

### Weaknesses

*External validity*: The cross-sectional validation involved patients attending only one dental teaching hospital in the north of England. Thus the measure requires further testing in a variety of environments, including specialist orthodontic practice. It would also be helpful to test the measure in a wider range of ethnic groups.

*Responder bias*: This is the phenomenon where responders answer questions in the way that they believe the researcher wants them to answer, rather than according to their own beliefs. Although participants were made aware at recruitment that their answers would not affect any future orthodontic treatment, it may still have had a subconscious effect and this may have influenced their answers. Another factor is the presence of significant other family members and parents/guardians when completing the questionnaire. Although specifically asked to complete the questionnaire on their own in a non-clinical environment, participants may not have been left alone to do so, or may not have wanted to complete it on their own and sought help from adults or siblings. Again, this may have affected their responses and questionnaire scores.

### Suggestions for further research

The measure needs testing in other primary and secondary orthodontic care settings to further evaluate cross-sectional validity. It also needs to be applied longitudinally to determine the responsiveness or ability to detect change over time.

Different modes of administration should be investigated. Traditional paper-based questionnaires can have problems with production, as evidenced by the printing error in this study, as well as environmental costs, time required for scoring/data inputting and security of data once collected. They may also be returned unanswered or incomplete, missing crucial information. An electronic platform, such as a computer, personal digital assistant or smartphone app, would enable easier distribution of the measure (especially if internet based), have a smaller environmental impact and eliminate incomplete entries and manual inputting of data, reducing potential errors. Responders may also find completion of electronic data entry easier. A disadvantage may lie in ensuring the security and confidentiality of data, but undoubtedly this can be overcome with the use of appropriate techniques.

## Conclusions

- Part 2 of this report has described the questionnaire formatting and cross-sectional evaluation of a new condition-specific measure for young people with malocclusion (MIQ);
- Rasch analysis was undertaken to reduce the number of items from the original 37 identified by qualitative inquiry to 17 questions, which resulted in a unidimensional scale free from DIF;
- The criterion and construct validity, internal reliability/consistency and test–retest reliability of MIQ were shown to be good;
- Further testing is required to assess generalisability and responsiveness.

## Disclaimer statements

**Contributors**: Philip Benson was responsible for the study design, ethical approval, data collection, analysis and interpretation, as well as the writing of the report. Susan Cunningham was responsible for the data analysis and interpretation and preparation of the report. Nahush Shah was responsible for the data collection, analysis and interpretation, as well as the preparation of the report. Fiona Gilchrist was responsible for the data analysis and interpretation, as well as the preparation of the report. Sarah Baker was responsible for the study design, data analysis and interpretation, as well as the preparation of the report. Samantha Hodges was responsible for the data analysis and interpretation, as well as the preparation of the report. Zoe Marshman was responsible for the study design, data analysis and interpretation, as well as the preparation of the report. All the authors have seen and approved the final report. Philip Benson is the guarantor.

**Funding**: Internal funding from the Orthodontic Department, University College London Eastman Dental Institute and Academic Unit of Oral Health and Development, School of Clinical Dentistry, University of Sheffield.

**Conflicts of interest:** None,

**Ethics approval:** The proportionate review sub-committee of North East – Sunderland Research Ethics Committee (REC Reference 11/NE/0359).
